# Effectiveness and safety of anti-CGRP monoclonal antibodies in patients over 65 years: a real-life multicentre analysis of 162 patients

**DOI:** 10.1186/s10194-023-01585-2

**Published:** 2023-06-02

**Authors:** Albert Muñoz-Vendrell, Sergio Campoy, Edoardo Caronna, Alicia Alpuente, Marta Torres-Ferrus, Candela Nieves Castellanos, Marina Olivier, Jaume Campdelacreu, Joan Prat, Javier Camiña Muñiz, Francisco José Molina Martínez, Ane Mínguez-Olaondo, Marta Ruibal Salgado, Sonia Santos Lasaosa, María Pilar Navarro Pérez, Noemí Morollón, Alba López Bravo, Luis Miguel Cano Sánchez, Sonia María García-Sánchez, Jésica García-Ull, Laura Rubio-Flores, Alicia Gonzalez-Martinez, Sonia Quintas, Ana Echavarría Íñiguez, Sendoa Gil Luque, María Victoria Castro-Sánchez, Vanesa Adell Ortega, Jessica García Alhama, Nuria Berrocal-Izquierdo, Robert Belvís, Samuel Díaz-Insa, Patricia Pozo-Rosich, Mariano Huerta-Villanueva

**Affiliations:** 1 Neurology Department, Hospital Universitari de Bellvitge-IDIBELL, Universitat de Barcelona, L’Hospitalet de Llobregat, Carrer de La Feixa Llarga S/N, 08907 Barcelona, Spain; 2 Neurology Department, Hospital de Viladecans-IDIBELL, Viladecans, Barcelona, Spain; 3 Headache Unit, Neurology Department, Hospital Universitari Vall d’Hebron, Barcelona, Spain; 4 Headache and Neurological Pain Research Group, Vall d’Hebron Research Institute, Department of Medicine, Universitat Autònoma de Barcelona, Barcelona, Spain; 5 Unidad de Cefaleas. Servicio de Neurología, Hospital Universitari I Politécnic La Fe, Valencia, Spain; 6 Unidad de Cefaleas. Servicio de Neurología, Hospital Universitari Son Espases, Palma, Spain; 7 Consulta Monográfica de Cefaleas. Clínica Rotger Quirónsalud, Palma, Spain; 8 Neurology Department, Donostia University Hospital-OSAKIDETZA, San Sebastián, Spain; 9 ATHENEA Neuroclinics, Policlínica Gipuzkoa Grupo Quironsalud, Donostia, Spain; 10 Department of Medicine, Faculty of Health Sciences, University of Deusto, Bilbao, Spain; 11 Neuroscience Area, Biodonostia Research Institute, San Sebastián, Spain; 12 Unidad de Cefaleas. Servicio de Neurología, Hospital Clínico Universitario Lozano Blesa, IIS Aragon, Zaragoza, Spain; 13 Unidad de Cefaleas Y Neuralgias. Servicio de Neurología. Hospital de La Santa Creu I Sant Pau, Barcelona, Spain; 14 Unidad de Cefaleas. Sección de Neurología, Hospital Reina Sofía. Instituto de Investigación Sanitaria de Aragón (IIS-A), Tudela, Spain; 15 Servicio de Neurología. Hospital Sant Joan Despí. Consorci Sanitari Integral, Sant Joan Despí, Spain; 16 Unidad de Cefaleas. Servicio de Neurología, Hospital Clínico Universitario de Valencia, Instituto de Investigación Sanitaria INCLIVA, Valencia, Spain; 17 Servicio de Neurología. Hospital Universitario General de Villalba, Madrid, Spain; 18 Headache Unit, Neurology Department, Hospital Universitario de La Princesa E Instituto de Investigación Sanitaria Princesa (IIS-Princesa), Madrid, Spain; 19 Unidad de Cefaleas, Hospital Clínico Universitario de Valladolid, Valladolid, Spain; 20 Servicio de Neurología. Hospital Universitario de Burgos, Burgos, Spain; 21 Servicio de Neurología. Hospital Carlos Haya de Málaga, Málaga, Spain; 22 Servei de Neurologia. Consorci Sanitari de L’Alt Penedès-Garraf, Barcelona, Spain; 23 Parc Sanitari Sant Joan de Déu. Servicio de Neurología. Sant Boi de Llobregat, Barcelona, Spain

**Keywords:** Calcitonin gene-related peptide, Monoclonal antibodies, Migraine, 65 years old, Real-world

## Abstract

**Background:**

Anti-CGRP monoclonal antibodies have shown notable effectiveness and tolerability in migraine patients; however, data on their use in elderly patients is still lacking, as clinical trials have implicit age restrictions and real-world evidence is scarce. In this study, we aimed to describe the safety and effectiveness of erenumab, galcanezumab and fremanezumab in migraine patients over 65 years old in real-life.

**Methods:**

In this observational real-life study, a retrospective analysis of prospectively collected data from 18 different headache units in Spain was performed. Migraine patients who started treatment with any anti-CGRP monoclonal antibody after the age of 65 years were included. Primary endpoints were reduction in monthly migraine days after 6 months of treatment and the presence of adverse effects. Secondary endpoints were reductions in headache and medication intake frequencies by months 3 and 6, response rates, changes in patient-reported outcomes and reasons for discontinuation. As a subanalysis, reduction in monthly migraine days and proportion of adverse effects were also compared among the three monoclonal antibodies.

**Results:**

A total of 162 patients were included, median age 68 years (range 65–87), 74.1% women. 42% had dyslipidaemia, 40.3% hypertension, 8% diabetes, and 6.2% previous cardiovascular ischaemic disease. The reduction in monthly migraine days at month 6 was 10.1 ± 7.3 days. A total of 25.3% of patients presented adverse effects, all of them mild, with only two cases of blood pressure increase. Headache and medication intake frequencies were significantly reduced, and patient-reported outcomes were improved. The proportions of responders were 68%, 57%, 33% and 9% for reductions in monthly migraine days ≥ 30%, ≥ 50%, ≥ 75% and 100%, respectively. A total of 72.8% of patients continued with the treatment after 6 months. The reduction in migraine days was similar for the different anti-CGRP treatments, but fewer adverse effects were detected with fremanezumab (7.7%).

**Conclusions:**

Anti-CGRP mAbs are safe and effective treatments in migraine patients over 65 years old in real-life clinical practice.

**Graphical Abstract:**

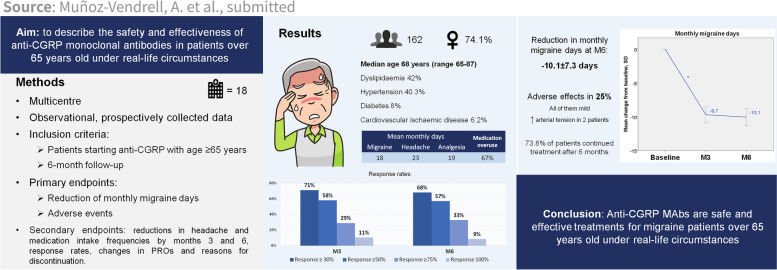

**Supplementary Information:**

The online version contains supplementary material available at 10.1186/s10194-023-01585-2.

## Introduction

Monoclonal antibodies (mAbs) targeting calcitonin gene-related peptide (CGRP) or its receptor have undeniably led to a new era for the treatment of migraine, given their generally fast and sustained effect and high responder rates [1]. The tolerability and safety of these treatments have been proven in diverse studies [2–4]. For this reason, its prescription in clinical practice is wide, although restricted in patients with previous cardiovascular or cerebral ischaemic events. However, data on anti-CGRP mAb use in elderly patients are still lacking, as clinical trials have implicit age restrictions and real-world evidence are still scarce.

Phase-3 trials of all four anti-CGRP mAbs included patients from 18 to 65 years old, with some exceptions. Both HALO studies [5, 6] and the FOCUS trial [7] with fremanezumab included patients up to 70 years old, and the PROMISE-1 trial [8] with eptinezumab included patients up to 75 years old. Post hoc analyses of these studies showed comparable treatment efficacy and safety in patients over 50 [9] and 60 years old [10]. The CONQUER trial with galcanezumab also included 29 patients from 65 to 75 years old (13 of them were enrolled in galcanezumab), with no meaningful differences in their clinical outcomes [11]. Nonetheless, clinical trials excluded patients with certain comorbidities (including cardiovascular risk factors or psychiatric disorders) and clinical characteristics (such as the presence of daily headache or medication overuse), thus raising the need for real-life studies to confirm similar results in other subsets of patients.

A recent study compared the effectiveness and safety of erenumab in real-life in patients over and under 65 years old, with similar reported outcomes, but was limited to a small sample of 15 patients per group [12].

In the present study, we aimed to evaluate the effectiveness and safety of anti-CGRP mAbs in migraine patients over 65 years old in a real-life multicentric cohort in Spain.

## Methods

### Patients

In this observational retrospective study of prospectively collected data from 18 different Spanish headache centres, we included consecutive patients starting any of the three commercialized anti-CGRP mAbs (erenumab, galcanezumab or fremanezumab) from December 2019 to June 2022 with the diagnosis of migraine according to ICHD-3 criteria [13] and who were over 65 years old by the date of initiation. According to the required criteria by the Spanish government for anti-CGRP mAb funded prescription, all recruited patients presented more than 8 monthly migraine days, with failure of at least 3 migraine preventive medications, including onabotulinum toxin A (BTX-A) for chronic migraine [14].

Anti-CGRP mAbs were administered monthly (erenumab 70 or 140 mg, galcanezumab 120 mg + 240 mg for the loading dose, or fremanezumab 225 mg) or quarterly (fremanezumab 675 mg) by subcutaneous injection. Each treatment was decided based upon individual patient characteristics at the discretion of the attending physician, as well as the association of concomitant oral preventive treatments or BTX-A.

### Outcome measures

All clinical data were prospectively collected at each centre from the moment of anti-CGRP initiation, with quarterly scheduled visits and a minimum follow-up of 6 months. The presence of comorbidities at baseline was retrospectively extracted from clinical records. The following variables were collected: age, sex, time since migraine diagnosis, episodic or chronic migraine and time since chronification, presence of aura, previous migraine preventive treatments, concomitance of oral preventive treatments or BTX-A injections and presence of comorbidities (cardiovascular risk factors, peripheral artery disease, cerebrovascular or cardiac ischaemic disease, obstructive sleep apnoea, chronic obstructive pulmonary disease, liver disease, nephropathy, psychiatric disorders or others). Clinical variables that were collected quarterly included the following: monthly migraine days (MMD), monthly headache days (MHD), frequency of monthly headache days by maximum intensity of pain (in a 4-point scale: none, mild, moderate or severe), monthly acute medication intake (MAMI), Headache Impact Test (HIT-6), Migraine Disability Assessment test (MIDAS), Patients’ Global Impression of Change (PGIC) scale (except in the baseline visit), presence of adverse effects and treatment discontinuation reasons. Headache parameters (MMD, MHD, MAMI and headache days by maximum intensity) were collected with standardized paper or electronic headache diaries brought by the patients on each clinical appointment. A headache day was defined as any calendar day with a documented headache episode. A migraine day was defined as a day with a headache that lasts at least 4 h and meets ICHD-3 criteria for migraine or probable migraine [13]; or a day with a headache that is successfully treated with a triptan, ergotamine, or other migraine-specific acute medication.

The primary endpoints included the reduction in MMD after 6 months of treatment and the presence of adverse effects. The secondary endpoints included the reduction in MMD after 3 months, the reduction in MHD and MAMI after 3 and 6 months, the change in frequency of days by intensity, the 30%, 50%, 75% and 100% response rates, changes in the HIT-6, MIDAS and PGIC scale scores, and the reasons for treatment discontinuation during the 6-month follow-up (due to inefficacy or adverse events). As a subanalysis of the cohort, the reduction in monthly migraine days and the proportion of adverse effects were also compared among the three monoclonal antibodies and between patients with and without concomitant oral treatment, concomitant BTX treatment and medication overuse at baseline.

### Statistical analysis

Primary and secondary endpoints were assessed using a descriptive analysis. Categorical variables were presented as absolute frequencies. Demographic and clinical variables were presented as medians and ranges or means and standard deviations according to the distribution. The normality of the distribution of each variable was evaluated with Kolmogorov–Smirnov and Shapiro–Wilk tests. Changes in mean MMD, MHD and MAMI were assessed by paired sample t-test. Differences in MMD and the proportion of adverse effects between the different mAbs were assessed by ANOVA. Differences in MMD and the proportion of adverse effects between patients with and without medication overuse, concomitant oral treatment and concomitant BTX-A treatment were assessed by chi square test or independent sample t-test as appropriate. A sensitivity analysis using the “last observation carried forward” technique was performed for quantitative endpoints at month 6, as some patients discontinued treatment after month 3.

The results of all the statistical analyses were interpreted with confidence intervals of 95% and a significance level of 5%. Statistical analyses were performed in SPSS v.20 (SPSS Inc., Chicago, USA). No statistical power calculation was conducted prior to the study. The sample size was based on the available data from the participating centres.

### Ethics approval and consent to participate

The study was approved by the Ethical Committee of the coordinating centre with reference EOM030/22. The confidential information of the patients was handled in accordance with Spanish regulations.

## Results

A total of 162 patients were included. Of these, 28 were lost to follow-up after discontinuing treatment at month 3 due to inefficacy or intolerance (these patients were not included in the analysis of frequency variables at month 6 but were included as non-responders in the global analysis at month 6; a sensitivity analysis of these frequency variables by “last observation carried forward” was performed to validate these results, see Appendix 1).

The median age was 68 years (range 65–87), and 74.1% of the patients were women. A total of 80.9% had chronic migraine, and 13% had aura. The median migraine onset age was 18 (IQR 14–26.5), and the time since chronification in cases of chronic migraine was 120 months (IQR 24.8–185). The patients’ comorbidities are detailed in Table 1. Previous and concomitant treatments are detailed in Table 2. At baseline, 37 patients (22.9%) were taking more than one oral concomitant treatment. A total of 23.5% of patients were treated with erenumab, 52.5% with galcanezumab, and 24.1% with fremanezumab (29 [17.9%] with monthly dose, and 10 [6.2%] with quarterly dose).

**Table 1:** Patients’ reported comorbidities at baseline

Comorbidity	n (%)
Dyslipidaemia	68 (42.0)
Hypertension	70 (40.3)
Anxiety	53 (32.7)
Depression	53 (32.7)
Fibromyalgia	16 (9.9)
Smoking	15 (9.3)
Diabetes	13 (8.0)
Obstructive sleep apnoea	12 (7.4)
Hypothyroidism	11 (6.8)
Tumours	10 (6.2)
Chronic obstructive pulmonary disease	7 (4.3)
Peripheral artery disease	5 (3.1)
Liver disease	5 (3.1)
Nephropathy	5 (3.1)
Cardiac ischaemic disease	4 (2.5)
Cerebral ischaemic disease	1 (0.6)
Others	31 (19.1)

**Table 2:** Previous and concomitant preventive treatments at baseline

Medication	Previous [n (%)]	Concomitant [n (%)]
Topiramate	138 (85.2)	12 (7.4)
Beta-blocker	117 (72.2)	33 (20.4)
Amitriptyline	140 (86.4)	25 (15.4)
Flunarizine	100 (61.7)	1 (0.6)
Anti-hypertensive	69 (42.6)	36 (22.2)
Onabotulinum toxin A	141 (87.0)	31 (19.1)
Others	103 (63.6)	44 (27.2)

**Fig. 1:**
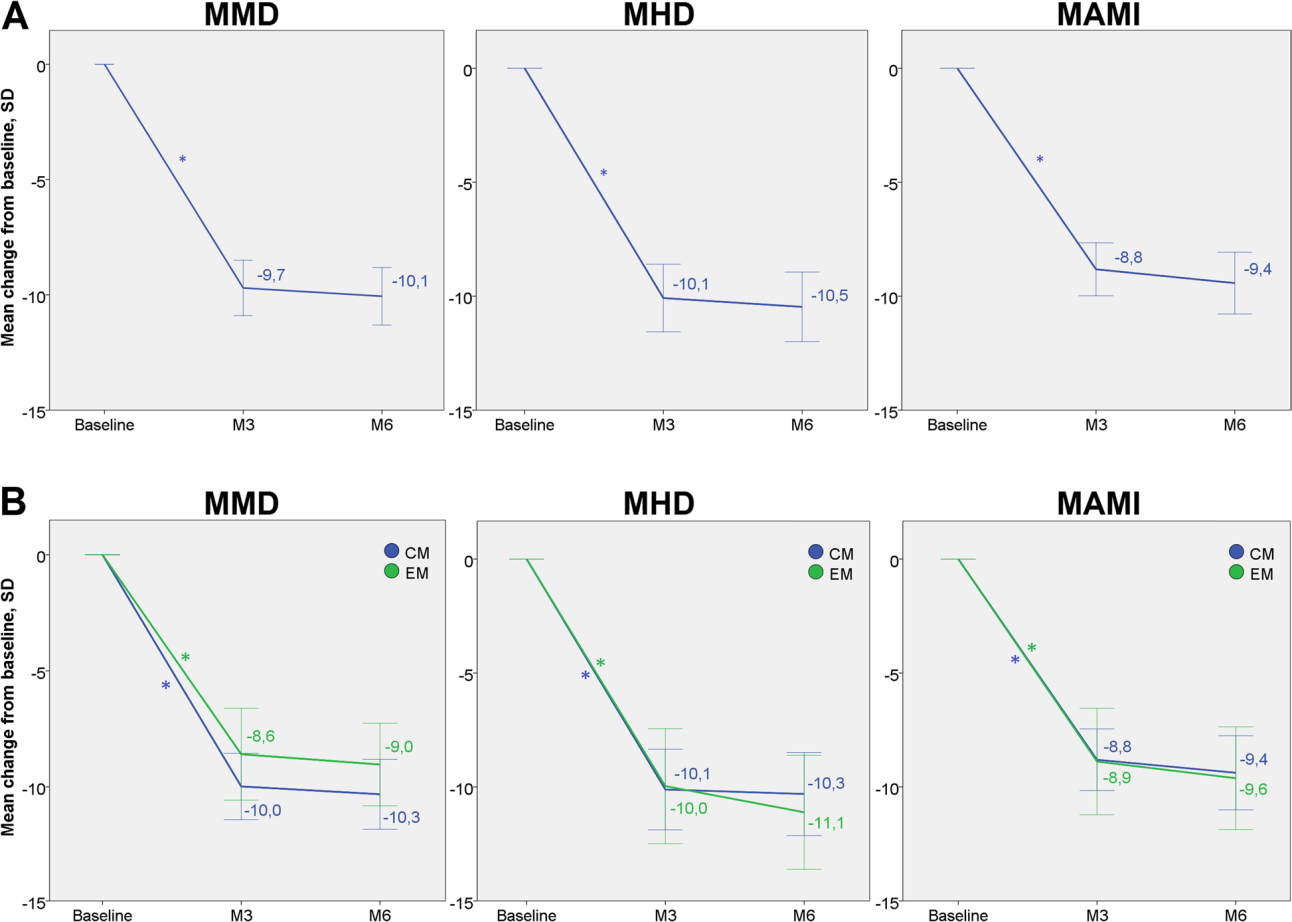
Change in MMD, MHD and MAMI after 3 (M3) and 6 months (M6). **A** Global mean changes in the entire cohort. **B** Mean changes by diagnosis at baseline: chronic migraine (CM) or episodic migraine (EM). *p < 0.001. Confidence intervals of 95%. MMD = monthly migraine days; MHD = monthly headache days; MAMI = monthly acute medication intake; SD = standard deviation, M3 = month 3; M6 = month 6; CM = chronic migraine; EM = episodic migraine

**Fig. 2:**
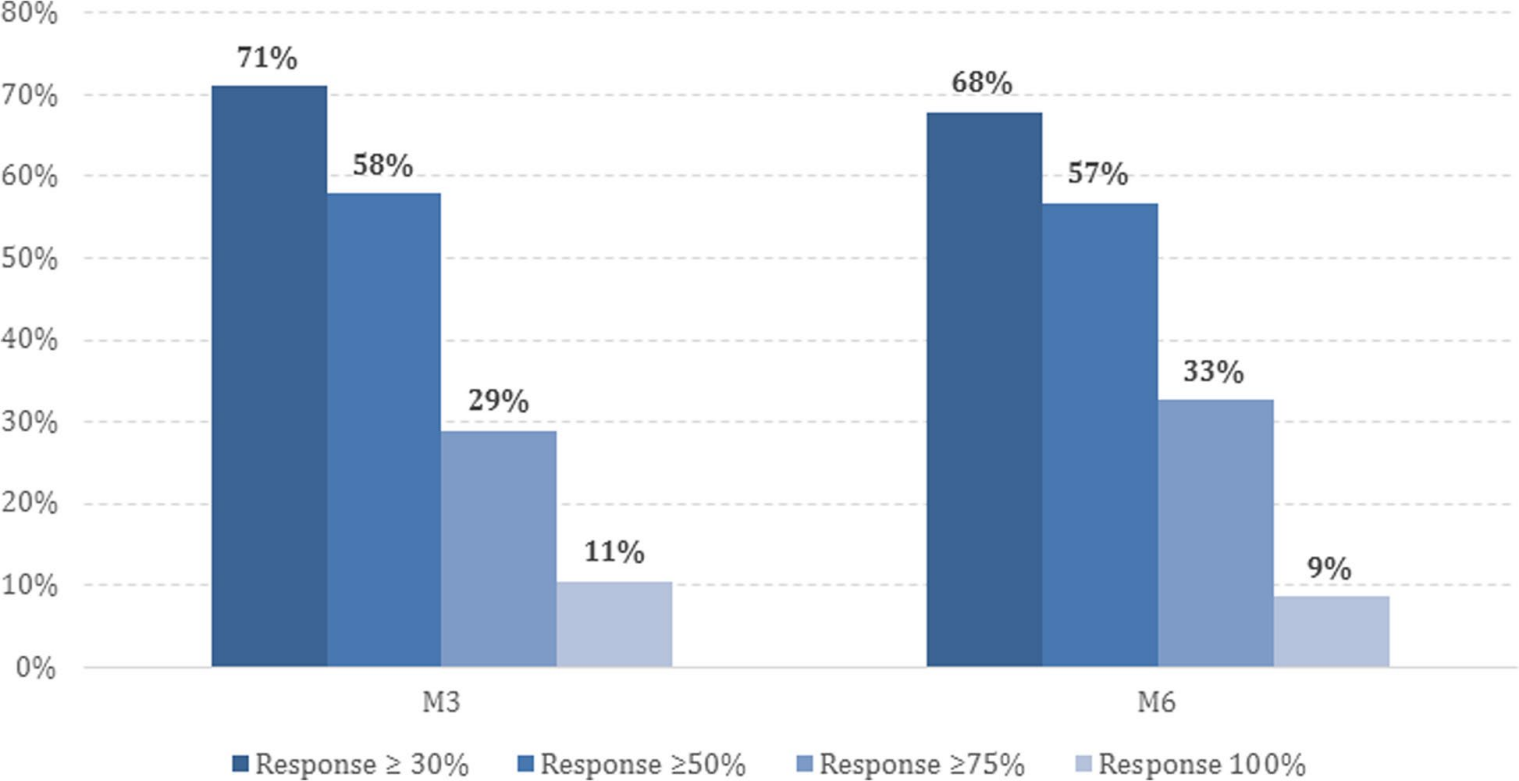
Proportion of ≥ 30%, ≥ 50%, ≥ 75% and 100% responders after 3 (M3) and 6 months (M6). M3 = month 3; M6 = month 6

Clinical characteristics at baseline and during the follow-up are detailed in Table 3. The reduction in MMD at 6 months was -10.1 ± 7.3 days (*p* = 0.0001). Forty-one patients (25.3%) presented adverse effects at some point during the follow-up, which are detailed in Table 4. The reduction in MMD after 3 months and the reduction in MHD and MAMI after 3 and 6 months were also significant (see Fig. 1). The proportion of responders is shown in Fig. 2. After clinical evaluation at month 6, 118 patients (72.8%) continued with the treatment and 44 patients (27.2%) discontinued, 36 (22%) due to inefficacy and 8 (4.9%) due to adverse effects. Treatment was discontinued in 28 patients (17.3%) at month 3 (20 due to inefficacy and 8 due to adverse effects: 1 injection site reaction, 1 incident of hypertension, 1 incident of migraine worsening, 1 incident of hypotension, 1 incident of transient facial erythema, 1 incident of general discomfort, 1 incident of unsteadiness and 1 incident of dizziness) and in 16 patients (9.9%) at month 6 (all due to inefficacy). At month 3, patients who discontinued anti-CGRP MAbs were taking erenumab in 4 cases (all due to inefficacy), galcanezumab in 19 (13 due to inefficacy, 6 due to adverse effects), and fremanezumab in 5 (3 due to inefficacy, 2 due to adverse effects). At month 6, patients who discontinued treatment were taking erenumab in 7 cases, galcanezumab in 7, and fremanezumab in 2 (all due to inefficacy).

**Table 3:** Clinical characteristics at baseline, at month 3 (M3) and at month 6 (M6)

	Baseline	M3	M6
N	162	162	134
MMD [mean ± SD]	18.0 ± 7.5	9.8 ± 9.0	7.3 ± 7.6
MHD [mean ± SD]	23.3 ± 6.9	15.0 ± 10.5	12.5 ± 10.0
Intensities [mean ± SD]	*n* = 113/162	*n* = 114/162	*n* = 96/134
Mild days/month	4.3 ± 6.3	6.1 ± 8.4	6.0 ± 8.4
Moderate days/month	8.3 ± 7.5	4.9 ± 6.2	3.4 ± 4.3
Severe days/month	9.3 ± 8.1	2.9 ± 5.4	2.2 ± 4.1
MAMI [mean ± SD]	18.9 ± 8.0 *n* = 153/162	11.1 ± 9.3 *n* = 147/162	9.4 ± 8.9 *n* = 129/134
Medication overuse [n (%)]	107 (66.5)	57 (35.2)	37 (27.8)
HIT-6 [mean ± SD]	65.9 ± 7.8 *n* = 141/162	57.1 ± 11.9 *n* = 135/162	55.2 ± 11.1 *n* = 114/134
MIDAS [mean ± SD]	80.3 ± 71.3 *n* = 129/162	45.2 ± 62.0 *n* = 121/162	34.4 ± 48.1 *n* = 101/134
PGIC [mean ± SD]	-	3.78 ± 2.0 *n* = 130/162	4.13 ± 2.1 *n* = 110/134
Concomitant oral treatment [n (%)]	102 (63.0)	89 (55.3)	64 (47.8)
Concomitant onabotulinum toxin A [n (%)]	31 (19.1)	25 (15.5)	18 (13.4)

*MMD* monthly migraine days, *SD* standard deviation, *MHD* monthly headache days, *MAMI* monthly acute medication intake

Missing data is shown below each variable

**Table 4:** Adverse effects during the follow-up

Adverse effect [n (%)]	M3 (*n* = 162)	M6 (*n* = 134)
Any	**33 (20.4)**	**19 (14.2)**
Constipation	16 (9.9)	16 (9.9)
Injection site reaction	6 (3.7)	1 (0.6)
Dizziness	3 (1.8)	1 (0.6)
Hypertension	2 (1.2)	0
General discomfort	2 (1.2)	1 (0.6)
Unsteadiness	1 (0.6)	0
Hypotension	1 (0.6)	0
Headache worsening	1 (0.6)	0
Transient facial erythema	1 (0.6)	0

Baseline characteristics and the reduction in MMD at month 6 were similar for the three different anti-CGRP mAbs, but the proportion of adverse effects was significantly lower for fremanezumab (see Table 5). Constipation was more frequent for erenumab (54.5% versus 40.9% for galcanezumab and 4.5% for fremanezumab), and injection site reaction was observed only in 7 patients who were given galcanezumab.

**Table 5:** Baseline characteristics and effectiveness and safety outcomes between different anti-CGRP mAbs

	Erenumab	Galcanezumab	Fremanezumab	*p*
n	38	85	39	
Baseline differences				
Age [mean ± SD]	69.8 ± 5.0	69.8 ± 4.4	68.8 ± 3.7	0.485
Proportion of chronic migraine [n (%)]	29 (76.3)	70 (82.4)	32 (82.1)	0.721
MMD [mean ± SD]	19.5 ± 7.9	17.6 ± 7.2	17.2 ± 7.7	0.365
MHD [mean ± SD]	22.8 ± 7.1	22.7 ± 7.1	24.9 ± 6.1	0.228
Medication overuse [n (%)]	27 (71.1)	56 (65.9)	24 (63.2)	0.760
MIDAS [mean ± SD]	73.3 ± 66 *n* = 33/38	89.5 ± 79 *n* = 68/85	66.2 ± 54.7 *n* = 28/39	0.284
Concomitant onabotulinum toxin A [n (%)]	5 (13.2)	20 (23.5)	6 (15.4)	0.322
Primary endpoints				
MMD reduction at M6 [mean ± SD]	-9.9 ± 8.2 *n* = 34/38	-10.4 ± 7.3 *n* = 66/85	-9.6 ± 6.5 *n* = 34/39	0.890
Total adverse effects [n (%)]	15 (39.5)	23 (27.1)	3 (7.7)	**0.005**

*MMD* monthly migraine days, *M6* month 6, *SD* standard deviation

Missing data is shown below each variable

The reduction in MMD at M6 was not significantly different between patients with medication overuse at baseline and those without (-10.4 ± 8.2 days vs. -9.4 ± 5.2 days respectively, *p* = 0.459), nor the proportion of adverse effects (25.2% vs. 24.1% respectively, *p* = 0.872). Similarly, no difference was observed in the reduction in MMD at M6 between patients with concomitant oral medication at baseline and those without (-9.6 ± 7.5 days vs. -10.8 ± 7.0 days respectively, *p* = 0.335), and neither in the proportion of adverse effects (27.5% vs. 21.7% respectively *p* = 0.414). Regarding concomitant BTX-A use at baseline, no difference was observed in reduction in MMD at M6 between patients with and without concomitant BTX-A use (-8.1 ± 5.8 vs. -10.5 ± 7.6 respectively, *p* = 0.288), nor in the proportion of adverse effects (22.6% vs. 26.0% respectively, *p* = 0.698).

There were no missing data on MMD, MHD, medication overuse and concomitant treatments. Data on MAMI, monthly headache frequency categorized by intensities, and HIT-6, MIDAS and PGIC scales were not available for all patients; therefore, those patients were not included in the calculation of global means for each of those variables at each visit. Missing data for each variable is shown in Tables 3, 4 and 5. There were no other missing data.

## Discussion

The results of this study revealed a significant reduction in headache frequency after 6 months of anti-CGRP mAb treatment in migraine patients over 65 years old (-10.1 ± 7.3 days in MMD and -10.5 ± 8.9 days in MHD), with ≥ 30%, ≥ 50%, ≥ 75% and 100% responder rates at month 6 of 68%, 57%, 33% and 9%, respectively. Patient-reported outcomes were also improved by month 6, including HIT-6 (65.9 ± 7.8 to 55.2 ± 11.1) and MIDAS (80.3 ± 71.3 to 34.4 ± 48.1) scores. These results are similar to previously reported outcomes in the general population in real-life [15–17].

Medication intake was also significantly reduced by month 6 (-9.2 ± 7.6 days in MAMI), with a prominent decrease in medication overuse rates (66.5% at baseline compared to 27.8% at month 6). These findings support the effectiveness of anti-CGRP mAbs in migraine patients with medication overuse, as previously suggested [18, 19]. This point is of special interest in the present cohort of older patients, assuming they are at a higher risk of analgesic medication side effects due to higher comorbidities, including ischaemic events [20].

Oral preventive medications for the treatment of migraine have a high rate of adverse effects, especially in older patients, in whom polypharmacy is frequent and susceptibility to intolerance is higher [21]. Treatment with anti-CGRP mAbs is an excellent alternative, given its minimal risk of interactions [22] and its beneficial effect in reducing the use of oral preventive treatments, as shown in our series (63% of concomitant oral treatment reduced to 47.8% at month 6).

The safety of using anti-CGRP mAbs to treat patients over 65 years old is still uncertain, given that patients included in clinical trials were selected and real-world data are scarce. As expected, the real-life circumstances of our study revealed a high prevalence of comorbidities among the patients, including dyslipidaemia (42%), hypertension (40.3%), anxiety (32.7%), depression (32.7%) and even cardiovascular ischaemic disease (6.2%). Even so, the proportion of reported adverse effects was relatively low after 6 months of treatment (25.3%), without any ischaemic event or other serious complication detected during the follow-up, and comparable to what has been reported in the general population [2–4, 23]. Only 8 patients (4.9%) discontinued anti-CGRP treatment due to adverse effects. These data provide evidence of the safety of using anti-CGRP mAbs in a setting of elderly patients with increased cardiovascular risk factors and comorbidities.

Special mention should be given to 2 patients (1.2%) who presented hypertension after mAb initiation. One of them presented with a transient increase in their diastolic readings without previous arterial hypertension diagnosis after the first dose of galcanezumab, but he did not require any specific treatment and allowed continuation of galcanezumab treatment. The other, who already had a known diagnosis of hypertension, presented with two hypertensive crises on the same day of the first and second administration of monthly fremanezumab; while that patient didn’t suffer target organ damage, she required prompt antihypertensive rescue treatment, thus leading to fremanezumab discontinuation. Consequently, caution should arise when prescribing anti-CGRP in older patients with resistant arterial hypertension, and specific monitoring should be considered.

In the present study, the reduction in MMD was not different between the 3 different anti-CGRP mAbs. However, fewer adverse effects were observed in the fremanezumab group (7.7%) than in the erenumab (39.5%) and galcanezumab (27.1%) groups, limited to only 3 patients who presented with constipation, transient facial erythema and a hypertensive crisis. This finding must be interpreted with caution, as the number of patients per group is relatively low, and for now, published meta-analyses have failed to find consistent differences between anti-CGRP mAbs safety and tolerability [24–26]. A possible explanation for this phenomenon could reside in the fact that a considerable proportion of patients with fremanezumab received quarterly doses, which implies fewer absolute days of subcutaneous administration compared to galcanezumab, and therefore a lower probability of local reaction; and that a slightly different mechanism of action than erenumab (which binds to the CGRP receptor) seems to minimize the incidence of constipation [27]. A higher proportion of patients with galcanezumab were observed in the present cohort, which is probably explained by the exclusive authorization of this MAb in particular in some regions of Spain during the time of the study.

Some limitations must be considered for this study. First, follow-up was limited to 6 months after the first treatment with anti-CGRP mAbs, which can omit patients responding later or after a mAb switch, and those presenting delayed drug adverse events. Second, although all clinical follow-up variables were prospectively collected, the presence of baseline comorbidities was assessed retrospectively, which can lead to biases of information if some conditions were unnoticed or not registered in the clinical records, and the use and dosing of concomitant treatments was poorly controlled. Likewise, missing data in the prospectively collected variables and the loss of follow-up of the patients who discontinued treatment at M3 may reduce the reliability of the results, even with the sensitivity analysis, which only considers missing data due to treatment discontinuation. Finally, when creating different arms to compare outcomes between anti-CGRP treatments, the number of patients per group was relatively low, thus generating low statistical power. Further studies are needed to confirm these findings supporting the effectiveness and safety of anti-CGRP mAb use in older migraine patients.

## Conclusion

Anti-CGRP mAbs are safe and effective treatments for migraine patients over 65 years in real-life clinical practice. Treatment with fremanezumab had fewer adverse effects in our cohort. No differences were observed in patients with and without concomitant oral treatment, concomitant BTX treatment and medication overuse at baseline. Further studies are needed in different populations, with larger samples and with a greater inclusion of prospective variables to increase the robustness of our results.

## Supplementary Information

**Additional file 1: Appendix 1.** Sensitivity analysis of quantitative endpoints at month 6.

## Data Availability

The datasets used and/or analysed during this study are available from the corresponding author on reasonable request from any qualified investigator.
