# Supplementary material for: Effectiveness and safety of anti-CGRP monoclonal antibodies in patients over 65 years: a real-life multicentre analysis of 162 patients
Source: J Headache Pain. 2023 Jun 2;24(1):63. doi: 10.1186/s10194-023-01585-2 (PMC10236648; doi:10.1186/s10194-023-01585-2)
Supplement: Supplementary file 1 — Additional file 1: Appendix 1. Sensitivity analysis of quantitative endpoints at month 6. [file 10194_2023_1585_MOESM1_ESM.docx]

**Appendix 1. Sensitivity analysis of quantitative endpoints at month 6.**

Missing data is shown below each variable for both assessments (observed and LOCF)

| Variables at M6 [mean±SD] | Observed | LOCF | Difference (CI 95%) | |
| --- | --- | --- | --- | --- |
| MMD  MHD  MAMI  Intensities  Mild days/month  Moderate days/month  Severe days/month  HIT-6  MIDAS  PGIC | 7.3±7.6 n=134/134  12.5±10.0 n=134/134  9.4±8.9 n=129/134  6.0±8.4 n=96/134  3.4±4.3 n=96/134  2.2±4.1 n=96/134  55.2±11.1 n=114/134  34.4±48.1 n=101/134  4.13±2.1 n=110/134 | 9.5±9.4 n=162/162  14.7±10.8 n=162/162  10.8±10.0 n=151/162  6.0±8.1 n=114/162  4.6±5.9 n=114/162  3.3±5.9 n=114/162  56.5±11.5 n=137/162  41.0±52.9 n=124/162  4.0±2.0 n=132/162 | -2.2 (-4.1, -0.2)  -2.2 (-4.6, 0.2)  -1.4 (-3.6, 0.9)  0.0 (-2.2, 2.6)  -1.2 (-2.6, 0.2)  -1.1 (-2.4, 0.3)  -1.3 (-4.2, 1.5)  -6.6 (-20.0, 6.8)  0.1 (-0.4, 0.6) | 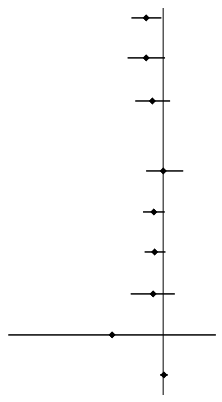 |

M6 = month 6; LOCF = last observation carried forward, MMD = monthly migraine days; MHD = monthly headache days; MAMI = monthly acute medication intake; SD = standard deviation.
